# Concepts from paediatric extracorporeal membrane oxygenation for adult intensivists

**DOI:** 10.1186/s13613-016-0121-0

**Published:** 2016-03-03

**Authors:** Warwick Butt, Graeme MacLaren

**Affiliations:** Paediatric Intensive Care Unit, Royal Children’s Hospital, Flemington Rd, Parkville, VIC 3052 Australia; Department of Paediatrics, University of Melbourne, Melbourne, Australia; Murdoch Children’s Research Institute, Clinical Sciences, Melbourne, Australia; Cardiothoracic Intensive Care Unit, National University Health System, 5 Lower Kent Ridge Rd, Singapore, 119074 Singapore

**Keywords:** Extracorporeal life support, Children, Critical care, Intensive care

## Abstract

Over the last 5 years, there has been a dramatic increase in the use of extracorporeal membrane oxygenation (ECMO) in adult patients with severe respiratory or cardiac failure. This contrasts to the use of the technology in neonatal and paediatric intensive care units, where it has been regarded as a standard of care for a number of conditions for over 25 years. Many innovations in ECMO circuitry or clinical management evolve first in one particular discipline and it may be helpful for individual clinicians to keep abreast of developments in ECMO across the entire age range, from neonatology to older adults. This review addresses nine concepts in ECMO that are better studied or established in paediatric medicine and considers their application in adult patients.

*Human nature will not change. In any future great national trial, compared with the men of this, we shall have as weak and as strong, as silly and as wise, as bad and as good. Let us therefore study the incidents in this as philosophy to learn wisdom from and none of them as wrongs to be avenged.* Abraham Lincoln, 1865

## Background

Patients started being treated with extracorporeal membrane oxygenation (ECMO) in the 1970s. Initial results were encouraging but associated with many complications. The publication of the first randomized control trial (RCT) of ECMO for acute respiratory distress syndrome (ARDS) in adults by Zapol et al. [1] showed no benefit and channelled efforts to improve the poor outcome for ARDS in other directions. However, Robert Bartlett from the University of Michigan decided to continue using ECMO in newborn infants with severe respiratory failure who could potentially recover more quickly than adults. This experience was quickly extended to older children and, in 1989, led to the establishment of the Extracorporeal Life Support Organization (ELSO) as a means of propagating knowledge about ECMO. Improved technology, equipment and understanding of patient and circuit pathophysiology led to a rapid increase in the use of ECMO for children. The use of ECMO for respiratory failure in adults continued in some centres during the 1990s but increased rapidly in the early 2000s and is now considered by many to be a standard therapy in many different clinical situations. The cumulative use of ECMO in patients is recorded by ELSO and reported biannually (Table 1). Over the last five years, there has been a dramatic increase in the use of ECMO in adults with respiratory or cardiac failure (Table 2). This explosion in the use of ECMO in adults is often attributed to the 2009 influenza A(H1N1) pandemic, but has also been due to ever-expanding indications in adult cardiac support, including post-cardiotomy shock, myocarditis, and as a bridge to ventricular assist device or thoracic organ transplantation. In 2012, more cases of cardiac ECMO were reported to ELSO in adults than in children. In 2013, the adult population similarly overtook the annual volume of paediatric and neonatal ECMO for respiratory indications.

**Table 1:** ELSO registry report: international summary (July 2015)

	Total patients	Survived ECLS (%)		Survived to DC or transfer (%)	
*Neonatal*					
Respiratory	28,217	23,791	84	20,978	74
Cardiac	6046	3750	62	2497	41
ECPR	1188	766	64	489	41
*Paediatric*					
Respiratory	6929	4579	66	3979	57
Cardiac	7668	5084	66	3878	51
ECPR	2583	1432	55	1070	41
*Adult*					
Respiratory	7922	5209	66	4576	58
Cardiac	6522	3661	56	2708	42
ECPR	1985	791	40	589	30
TOTAL	69,114	49,063	71	40,764	59

*ELSO* extracorporeal life support organization, *ECLS* extracorporeal life support, *ECPR* extracorporeal cardiopulmonary resuscitation, *DC* hospital discharge

**Table 2:** ELSO data on annual ECMO use 2010–2014 (July 2015)

	2010	2011	2012	2013	2014
*Respiratory*					
Neonatal	884	847	850	779	850
Paediatric	379	411	472	491	470
Adult	529	666	949	1423	1779
*Cardiac (patient age)*					
0–30 days	309	393	416	454	433
30 days–1 year	241	273	263	290	314
1–16 years	170	221	237	228	261
Over 16 years	423	597	1026	1235	1494

Many innovations in ECMO circuitry or clinical management evolve first in one particular discipline. For example, the first use of dual-lumen ECMO catheters, the first rigorous randomized controlled trial showing benefit to ECMO, and the most extensive long-term outcome studies were all performed in neonatal patients. We believe that it is helpful for individual clinicians to keep abreast of developments in ECMO across the entire age range, from neonatology to older adults. This review addresses nine concepts in ECMO that are better studied or established in paediatric medicine and considers their application in adult patients.

## Review

### ECMO is not proven, but it works

There are a number of excellent recent reviews that summarize the current clinical use of ECMO in critically ill adults with cardiopulmonary disease [2, 3] and also the evidence for either venoarterial [4] or venovenous ECMO [5, 6] in adults with primary cardiovascular or respiratory failure. It is interesting to consider that in an era of evidenced-based medicine and randomized controlled trials in critical care medicine, there are no studies that unequivocally prove the benefit of ECMO in adult patients. There are numerous studies that present outcomes of patients in many different ways, but all of these are designed to encourage the use of ECMO for cardiac and/or pulmonary failure. These methods of data presentation include:

1. A case series of patients with a particular problem who had good survival. These reports may suffer from publication bias.
2. A cohort compared to historical controls. These studies are usually positive because the newer treatments are better than the old ones.
3. A cohort compared to matched case controls or propensity analysis. This can be a useful attempt to evaluate efficacy for complex clinical situations in terms of severity of illness, disease and multiple complex treatments.
4. A cohort compared to the ELSO database. This will favour the cohort because the ELSO database is presented cumulatively over 26 years, has many inexperienced centres with the initial “learning curve” problems and includes complications of both ECMO and disease.
5. Finally, there are three RCTs [1, 7, 8] in adult respiratory failure. The outcomes have been interpreted in different ways, depending on the view of those in favour or opposed to ECMO (Table 3).

**Table 3:** Randomized controlled trials in adult ECMO

Author (ref no)	CMV (lived/total)	ECMO (lived/total)	Pro-ECMO	Anti-ECMO	Today’s perspective
Zapol et al. [1]	4/48 (8 %)	4/42 (10 %)	Change needed? Different patients with different diseases	Does not work	VV not used, ECMO duration arbitrarily limited to 5 days, ECMO started very late
Morris et al. [7]	8/19 (42 %)	7/21 (33 %)	2/3 death due to bleeding, learning curve	Does not work, protocols are better	Small study, outdated ECMO technology, training is important
Peek et al. [8]	41/87 (47 %)	57/90 (63 %)	ECMO works	Only 68 % received ECMO	Intention to treat analysis, ECMO in care plan probably beneficial but still unproven

*CMV* conventional mechanical ventilation, *ECMO* extracorporeal membrane oxygenation, *VV* venovenous, *CESAR* conventional ventilator support versus extracorporeal membrane oxygenation for severe adult respiratory failure

There have been many cohort studies published in newborn infants and children with respiratory or circulatory failure similar to the adult literature. Similarly, there have also been three RCTs published in children with respiratory failure (Table 4). Each one of these six RCTs has been subjected to criticisms of their methodology, such as problems with randomization, using outdated cannulation and ventilation strategies, and failure to adequately standardize treatment in the control arms. The last criticism was levelled at the most recent RCT in adult patients, the CESAR study [8], where patients in the control arm remained at their initial hospital and received whatever unstandardized care was provided to them, while those in the treatment arm were transported to a very experienced ECMO centre where the majority, not but all, received ECMO. Similar criticisms had been directed at the first successful RCT of ECMO in neonatal respiratory failure, conducted in the UK over a decade before [9]. It is interesting to reflect on what level of proof needs to be demonstrated in order to accept a new form of treatment or life support. In general, clinicians who are experienced with ECMO believe that its efficacy is self-evident and those who argue that there is insufficient evidence for it often have minimal experience with it. There is now more evidence supporting the use of ECMO for certain conditions than for many other forms of life support. Where are the RCTs of mechanical ventilation versus normobaric oxygen in ARDS, or inotropes versus placebo in septic shock?

**Table 4:** Randomized controlled trials in neonatal and paediatric ECMO

Author (ref no)	CMV (lived/total)	ECMO (lived/total)	Pro-ECMO	Anti-ECMO	Today
Bartlett et al. [52]	0/1 (0 %)	11/11 (100 %)	It works, novel study design to minimize death rate	Amazement that the study was approved by the review board	Play the winner, possible bias, small numbers
O’Rourke et al. [53]	6/10 (60 %)	28/29 (97 %)	It works	It might work but is unproven	Small numbers
UK neonatal study [9]	38/92 (41 %)	63/93 (68 %)	It works	No standardized protocol for treatment of control patients	Suggestive of benefit but some methodological problems

*CMV* conventional mechanical ventilation, *ECMO* extracorporeal membrane oxygenation, *UK* United Kingdom

The view of most neonatal and paediatric intensivists is that ECMO is a standard therapy that is part of clinical practice guidelines for critically ill children with diverse causes of cardiopulmonary failure. In a detailed review of recent evidence from 2002 to 2012 on the use of ECMO [10], the authors concluded, *Despite a large number of published studies there remains a paucity of high*-*quality clinical trials. The available data support the continued use of ECMO for respiratory failure refractory to conventional therapy for neonatal and pediatric patients without significant comorbidities. Further research is needed to better quantify the benefit of ECMO and the utility of many therapies applied to ECMO patients*. Another review [11] concluded *that continued examination of the criteria and circumstances where extracorporeal life support is applied as well as outcomes which include mortality, cost effectiveness and quality of life are needed areas of continued research.* In addition to these goals, further research should focus on the optimal timing of ECMO initiation, including examining the consequences of these decisions with detailed functional outcome studies.

### The systems that provide ECMO vary: all are valid and all have their problems

The establishment of an ECMO programme will often have a key individual who becomes the main proponent or champion for the use of the technology. While most paediatric programmes developed with a combination of neonatal physicians and general surgeons, some programmes developed from paediatric intensive care clinicians or cardiothoracic surgeons. This has led to different systems of care for patients, different clinical care protocols, equipment and cannulation techniques that vary dramatically across the world [12]. Peripheral cannulation may be percutaneous or surgical and removal of these cannulas may or may not involve surgery, with or without vascular reconstruction. Femoro-femoral cannulation has substantial risks of limb ischaemia, compartment syndrome and venous obstruction in children and adults, and much debate occurs about the best method of cannulation [13–18]. The choice of peripheral or central (transthoracic) cannulation also varies dramatically, even when cardiac surgeons perform the procedure. An example of this is at the Royal Children’s Hospital, Melbourne, Australia, where the majority of children who receive ECMO after cardiac surgery do so via central cannulation, while at East Midlands Congenital Heart Centre, Glenfield Hospital, Leicester, it is more common to cannulate through a jugular-carotid approach (G Peek, personal communication). There are greater risks of mediastinitis and reoperation for bleeding with central cannulation, whereas jugular-carotid cannulation is associated with a higher risk of neurological injury [19]. Similarly, there is a trend in some international centres caring for adult patients to move away from femoral cannulation and use more axillary arterial cannulation, which mitigates the risks of leg ischaemia and differential cyanosis, and facilitates extubation and ambulation on ECMO. The major downside to this approach is that it prolongs the time taken to cannulate and is not suitable during cardiac arrest. Ongoing reappraisal of the quality and methods of institutional care is important as new evidence becomes available.

### Configuration of cannulation should be physiologically based and may change over time

While each programme will have their method of cannulation and their preferred configuration whether for VV [5] or VA ECMO [20], these should have a sound physiological basis. An additional consideration in younger children relates to the size and development of the vasculature. ECMO cannulas in children occupy a greater proportion of the vessel cross-sectional area when compared to adults and, in infants, the femoral vessels are relatively hypoplastic, making cannulation technically difficult or practically impossible. This limits the number of possible cannulation sites and drove researchers to develop the first dual-lumen VV ECMO catheters for use in the internal jugular vein. As children begin to walk and develop their leg muscles, the vessels grow and become easier to cannulate. Moreover, during the course of an ECMO run, new clinical situations with changing pathophysiology may necessitate an additional or complete change of cannulas. In children, this is often conversion of VA to VV in pneumonia, once circulatory collapse has resolved over the first 3 days, or the need for left atrial decompression in left heart failure [21]. In adults, these include the change from a standard VV configuration to a VV-V, i.e. an extra drainage cannula, which will improve patient oxygen saturation or to V-VA, i.e. an extra arterial return catheter to augment circulatory function, which can be important in supporting the right ventricle during prolonged respiratory failure.

### Indications for ECMO are dynamic and change accordingly, albeit at different rates in different parts of the world

When ECMO started in children, the presence of active bacterial infection was considered a contraindication but some clinicians used ECMO for refractory septic shock regardless, and successful outcomes were first reported in the early 1990s [22]. Clinical practice guidelines and College recommendations in children with septic shock have included ECMO since 2002 [23]. The use of ECMO in adults with septic shock is now occurring in some centres [24, 25]. Likewise, ECMO was not recommended in children with leukaemia and immunosuppression until a review of the ELSO experience [26] and an accompanying editorial [27] demonstrated good patient outcomes. Since then, many children with leukaemia and solid tumours are considered for ECMO. In both children and adults, ECMO is often used to support cardiopulmonary function for early graft failure after heart or lung transplantation, with good results [28, 29].

As indications for initiating ECMO change, clinical management [2–6, 10–12] will also have to change. For example, if ECMO is used after cardiac surgery, bleeding will be more likely than if ECMO is not used. Younger children have immature coagulation systems, such as lower concentrations of antithrombin, and a greater propensity towards intracranial haemorrhage because of their fragile germinal matrices. They are thus more vulnerable to miscalculations in anticoagulation and blood product management. Clinicians change their practice to intensify monitoring of such values as anti-Xa and antithrombin levels. Similarly, adults after cardiopulmonary bypass have a greater risk of bleeding complications, not just because of recent surgery, but also because of the formation of antithrombin-heparin complexes, platelet dysfunction and release of endogenous glycosaminoglycans induced by the bypass circuit [30]. If ECMO is used in patients with sepsis and disordered coagulation, then anticoagulation will become more difficult and circuit thrombosis and patient haemorrhage is more likely.

### Goals of therapy are vital in effecting patient management and reducing complications

The principal goal of therapy with ECMO is to maximize quality of life and achieve patient survival with minimal complications. Targeting physiological goals may alter this. For example, a five-year-old has fulminant septic shock with refractory hypotension and progressive acidosis. ECMO is commenced. What blood flow should the patient receive from the ECMO circuit? If 120 mls/kg/min is chosen then this is achievable with peripheral cannulation; but if the goal of therapy is 150–200 mls/kg/min then sternotomy and central cannulation with large cannula are needed. 120 ml/kg/min may be insufficient in some children with distributive shock; thus, peripheral cannulation may expose the child to the risks of ECMO without the benefits. This may be the reason for the poor outcomes seen in some studies of ECMO for adult septic shock [24, 25]. Conversely, central ECMO may more easily achieve physiological goals but requires a dedicated cardiac surgical team and carries a greater risk of iatrogenic bleeding and infection.

Another example is a 20-year-old with severe pneumonia and single organ failure on VV ECMO and with SaO_2_ 80 %. All attempts to improve saturation fail. Some ECMO programmes would (in the presence of adequate haemoglobin, cardiac function and adequate perfusion) accept this, while others would insert an extra venous catheter to increase SaO_2_ to 90 %. Paediatric ECMO clinicians have long experience managing children with cyanotic congenital heart disease and may be prepared to accept lower systemic oxygen saturations providing the arterial-venous oxygen difference, arterial lactate and organ function parameters are acceptable. There are many other less dramatic differences in how patients are managed on ECMO due to the many different therapeutic goals such as fluid balance, blood pressure, nutrition, sedation and the team’s willingness to manage the patient with or without an endotracheal tube. Perhaps the most variation occurs in anticoagulation protocols of patients on ECMO because of differences in both monitoring and therapeutic goals.

### Large, committed ECMO programmes have better outcomes

Large congenital cardiac surgical centres use ECMO for routine mechanical circulatory support for low cardiac output after surgery. However, outcomes are better if there is an in-house surgeon capable of rapid cannulation [31] or if the ECMO is used early rather than delayed [32]. Not surprisingly, centres of excellence with large volumes have better patient results than centres with small volume of patients receiving ECMO [33, 34]. This effect appears particularly prominent for cardiac disease [33].

### Outcome assessment is essential

Knowledge of patient outcome is an essential part of critical care. This is especially relevant to ECMO. However, what appears as a simple question, “what is my patient’s outcome?” actually requires a very complex answer [35]. What outcome should we choose and when should we assess it? Patient survival is the easiest to assess but at discharge from ICU, discharge from hospital or some fixed time point after admission (e.g. 30 days) or 6–12 months after discharge? If we do a functional assessment, do we examine disease-specific or global-assessment outcomes? Do we use quality-of-life tools (generic or specific) and if so, from whose perspective? Do we do a telephone interview or examine the patient? Long-term outcomes have been part of neonatal and paediatric care for many years and outcomes are well known [36, 37]. Unfortunately, they reflect treatment protocols that no longer exist and have changed. In general, the easier the outcome measure is to assess, the less meaningful it may be. Although it is the common standard for ECMO, short-term survival to hospital discharge is not a particularly satisfactory outcome measure because not only can it not be used to assess quality of life in survivors, some studies have demonstrated that there is a significant late death rate in a number of conditions [38, 39]. More and more studies are being conducted examining very late outcomes after paediatric ECMO, ranging from median follow-up times of 4–15 years, and including a wide range of outcome measures such as long-term survival, neurodevelopmental outcomes and quality of life [38–44]. Several of these studies have no parallel in adult ECMO populations or, indeed, adult ICU populations and include testing exercise capacity 10–15 years after ECMO [40], 7-year neurocognitive follow-up after an RCT of ECMO [41] and 8-year nationwide follow-up of every neonatal ECMO survivor in a country [42].

### Haemolysis causes demonstrable harm

Haemolysis, as reflected by rising plasma haemoglobin, is associated with increased mortality in children [45]. Haemolysis may be more common in small children because of the technical difficulties associated with placing large cannulas in small blood vessels. There is sufficient evidence that iatrogenic haemolysis is a contributor to critical care mortality and every effort should be directed towards minimizing it [46]. In adults, persistently high plasma haemoglobin has a complex but important association with CRRT requirements, longer ECMO runs and higher mortality rates [47, 48].

## Complex congenital heart disease and ECMO is coming to your adult ICU soon

In many high-income countries, there are now more adults alive with congenital heart disease than children, including complex cyanotic congenital heart disease [49]. Recent data from the Australia and New Zealand Fontan Registry showed that patients who underwent the conventional (atriopulmonary) Fontan operation had 76 % 25-year survival. With more recent modifications (lateral tunnel or extracardiac conduit), current 10-year survival in these patients is 97 % [50]. These patients have undergone multiple operations, require more elaborate cannulation strategies to decompress both vena cavae and commonly have difficult vascular access and complex physiology not often understood by clinicians untrained in or unfamiliar with this population. Many of them are candidates for extracorporeal support, as a bridge to recovery (e.g. following surgery or as a result of concomitant respiratory infection), surgical revision (e.g. thrombosis of the conduits) or ventricular assist device implantation and transplantation (e.g. irreversible myocardial failure due to uncorrectable atrioventricular valve regurgitation and chronic volume overload). Early cannulation is often necessary because extracorporeal cardiopulmonary resuscitation (ECPR) in this patient group is associated with extremely poor outcomes, probably because of the ineffectiveness of conventional CPR at providing systemic blood flow while simultaneously causing cerebral injury because of impedance to cerebral venous drainage. Medium-term goals after cannulation may require extensive multidisciplinary collaboration because of the need to gather the requisite information in order to plan bridging the patient from ECMO to a more definitive strategy. Meticulous understanding of the Fontan circulation is essential to optimize outcomes. However, few countries have prepared adequately for the incoming wave of critically ill patients with adult congenital heart disease (ACHD) and it is likely that multidisciplinary collaboration between adult and paediatric clinicians experienced with these conditions offers the best hope at present for patient care, until sufficient dedicated ACHD institutions can be established [51].

## Conclusions

There are many unanswered questions about the role of ECMO in adults and children, but it is clear that, similar to mechanical ventilation, no classic RCT will be done. It is a therapy that has evolved disease-by-disease and patient group-by-patient group. With increasing safety and improving technology, it is a therapy that is here to stay. Indications will change, and the role of mechanical support as an individual therapy and a platform to facilitate other therapies is increasing. Paediatric clinicians have a long perspective on ECMO, outcome and follow-up, but adult clinicians are increasingly using ECMO in a variety of situations and rapidly developing new clinical paradigms of care. Good communication will ensure that both groups continue to learn from each other.
